# Characterization of a Broadly Reactive Anti-CD40 Agonistic Monoclonal Antibody for Potential Use as an Adjuvant

**DOI:** 10.1371/journal.pone.0170504

**Published:** 2017-01-20

**Authors:** Cameron Martin, Suryakant D. Waghela, Shehnaz Lokhandwala, Andy Ambrus, Jocelyn Bray, Christina Vuong, Vanitha Vinodkumar, Paul J. Dominowski, Sharath Rai, Duncan Mwangi, Dennis L. Foss, Waithaka Mwangi

**Affiliations:** 1 Department of Veterinary Pathobiology, Texas A&M University, College Station, Texas, United States of America; 2 Zoetis, Kalamazoo, Michigan, United States of America; The Ohio State University, UNITED STATES

## Abstract

Lack of safe and effective adjuvants is a major hindrance to the development of efficacious vaccines. Signaling via CD40 pathway leads to enhanced antigen processing and presentation, nitric oxide expression, pro-inflammatory cytokine expression by antigen presenting cells, and stimulation of B-cells to undergo somatic hypermutation, immunoglobulin class switching, and proliferation. Agonistic anti-CD40 antibodies have shown promising adjuvant qualities in human and mouse vaccine studies. An anti-CD40 monoclonal antibody (mAb), designated 2E4E4, was identified and shown to have strong agonistic effects on primary cells from multiple livestock species. The mAb recognize swine, bovine, caprine, and ovine CD40, and evoked 25-fold or greater proliferation of peripheral blood mononuclear cells (PBMCs) from these species relative to cells incubated with an isotype control (p<0.001). In addition, the mAb induced significant nitric oxide (p<0.0001) release by bovine macrophages. Furthermore, the mAb upregulated the expression of MHC-II by PBMCs, and stimulated significant (p<0.0001) IL-1α, IL6, IL-8, and TNF-α expression by PBMCs. These results suggest that the mAb 2E4E4 can target and stimulate cells from multiple livestock species and thus, it is a potential candidate for adjuvant development. This is the first study to report an anti-swine CD40 agonistic mAb that is also broadly reactive against multiple species.

## Introduction

Cluster of differentiation 40 (CD40) receptor, a member of the tumor necrosis factor superfamily, is expressed on B-cells, macrophages, dendritic cells (DCs), endothelial cells and fibroblasts [1, 2]. The CD40 is also expressed on several types of human cancer cells including bladder, breast, and ovarian [3, 4]. A natural ligand for CD40, CD40L (CD154), is expressed by activated CD4^+^ T-cells [3, 5]. The CD40L interacts with CD40 by crosslinking multiple CD40 molecules and thereby provides a critical signal for antigen presenting cell (APC) activation [6, 7]. The CD40-CD40L interaction stimulates B-cells to undergo somatic hypermutation, class switch recombination, clonal expansion, upregulation of major histocompatibility complex II (MHC-II) and secretion of proinflammatory cytokines. For example, humans suffering from X linked hyper-IgM syndrome are deficient in either CD40 or CD40L, and thus do not undergo class switch recombination or somatic hypermutation. The X-linked hyper IgM syndrome leads to high proportions of IgMs and low levels of IgA, IgE, and IgG present in the serum, absence of germinal centers, and the inability to mount a T-cell-dependent humoral response [8]. The interaction of CD40L with CD40 on macrophages, induces synthesis and release of nitric oxide, upregulation of MHC-II expression, and secretion of proinflammatory cytokines [9, 10].

Naive T-cells require two distinct signals from APCs for proper activation and induction of differentiation: signal 1 is provided by peptide antigens in the context of MHC molecules, while signal 2 is delivered by costimulatory molecules such as CD80 or CD86 present on DCs [11]. For antigen-loaded DCs to provide these signals effectively, they require activation to upregulate surface expression of MHC-peptide complexes and costimulatory molecules, and to secrete pro-inflammatory molecules such as IL-12 [12]. The DC activation is an innate response that adjuvants as well as live vaccines stimulate through pattern recognition receptor (PRR)ligand signaling, chemokine and cytokine secretion [13].

Expression of CD80/CD86 is upregulated by PRR ligands, TNF-α and IFN-γ, as well as interaction between CD40 on APCs and CD40L [11–13]. Even though the DCs from CD40^-/-^ or CD40L^-/-^ mice present antigens on MHC class I and II molecules and express high levels of CD80/86, CD4^+^ and CD8^+^ T cell immunity is not elicited [12]. This indicates that distinct CD40/CD40L signaling that functions together with antigen presentation and co-stimulation is required to generate functional CD4^+^ T-helper and CD8^+^-CTLs [12]. This signaling critically requires APC-T cell contact, CD40L expression, or an agonistic anti-CD40 antibody [14–16]. With regard to priming CD8^+^CTLs, DCs are first activated by CD4^+^ T-helper cells through CD40CD40L interactions and they in turn activate CD8^+^ CTLs by, in part, secreting proinflammatory cytokines, such as IL-12. The secreted IL-12 is a powerful inducer of IFN-γ production and Th1 differentiation [17]. More importantly, DC activation through CD40 signaling overcomes tolerance and may release immature DCs from the control of regulatory CD4^+^CD25^+^ T-cells [18]. Agonistic mAbs against CD40 directly mimic CD4^+^ T-cell help *in vivo* in response to T-cell dependent antigens [19–22]. Using CD40-targeted antigen delivery, up to 1000-fold increased antibody responses has been reported [22, 23]. *In vitro* stimulation of APCs using various forms of CD40 agonists like membrane-associated CD40L, soluble CD40L (sCD40L), or anti-CD40 antibodies evokes distinct functional responses [24]. Conjugation of an agonistic anti-CD40 mAb to a peptide based vaccine, a whole killed virus vaccine, or a commercially produced split influenza virus vaccine significantly enhanced antigen-specific antibody and T-cell responses [25]. Antibody class switching is also attributed to such agonistic anti-CD40 antibodies following interaction with CD40 on B-cells[26]. Therefore, CD40 activation using a high affinity agonistic antibody is an attractive strategy for adjuvant development. The livestock industry is an economically important sector that generates revenue and jobs globally, but disease control is partly hindered by lack of safe and effective adjuvants [27]. In addition, livestock serve as models for infectious and non-infectious human diseases. Therefore, development and optimization of an agonistic anti-CD40 mAb is likely to result in generation of new adjuvants for use in livestock [28, 29]. In this study, we developed an anti- swine CD40 mAb and characterized its agonistic activity on cells from livestock species.

## Materials and Methods

### Alignment of CD40 sequences

Swine (AAL92924.1), Bovine (NP_001099081.1), Ovine (NP_001068569.1), Caprine CD40 (XP_005688676.1), Human (NP_001241.1), Murine (AAH29254.1), Feline (XP_003983558.1), Canine (NP_001002982.1), Cavy (XP_013005826.1), and Erinaceine (XP_007533138.1) protein sequences were obtained from GenPept. Sequences were aligned using Jalview 2.9.0b2 and analyzed by Clustal X. Homology between these CD40 protein sequences was calculated by Protein BLAST to determine percentage identity to swine CD40 sequence.

### Cell culture

Hybridomas, human embryonic kidney (HEK)-293A cells, and fresh peripheral blood mononuclear cells (PBMCs) were grown in an atmosphere of 5% CO_2_ at 37°C. Dulbecco Modified Eagle Medium (DMEM) supplemented with 10% fetal bovine serum (FBS), 2mM GlutaMAX^™^, 0.01M HEPES, 100U/mL Penicillin-Streptomycin, 0.1M non-essential amino acids, 1mM sodium pyruvate, and 0.1mM 2-mercaptoethanol (2-ME) was used to grow hybridomas. HEK-293A cells were grown in DMEM supplemented with 10% FBS, 2mM GlutaMAX^™^, 0.01M HEPES, 0.1M non-essential amino acids, and 0.1M 2-ME. Swine, bovine, caprine, and ovine PBMCs were cultured in RPMI 1640 supplemented with 10% FBS, 2mM GlutaMAX^™^, 0.1M nonessential amino acids (0.1M), HEPES (0.01M), 0.1M 2-ME, and Penicillin-Streptomycin (100U/mL). FreeStyle™ 293-F cells were grown in Freestyle^™^ Expression Media (ThermoFisher) in an atmosphere of 8% CO_2_ at 37°C.

### Generation and purification of recombinant swine CD40

Total RNA was isolated from swine spleen using Trizol® (Invitrogen) and used for cDNA synthesis using Superscript II reverse transcriptase (Invitrogen). Sequences encoding full length swine CD40 (swCD40) or the extracellular domain (swCD40ED) was PCR amplified with Accuprime Pfx DNA Polymerase (Invitrogen) using primers based on GenBank sequence AF248545.1. The PCR product encoding the swCD40ED was ligated into PCR-TOPO vector (Invitrogen), and the ligation mix was used to transform *E*. *coli* TOP 10 cells (Invitrogen). Following colony screening and DNA sequencing of positive clones, the construct encoding the authentic swCD40ED was modified by overlap extension PCR to incorporate a secretory signal sequence at the 5’ terminus and the FLAG-tag at the 3’-terminus. The resultant gene encoding the swCD40ED and the PCR product encoding full length swCD40 were sub-cloned into the eukaryotic expression vector pcDNA3.1-TOPO (Invitrogen) and verified by sequencing. A construct encoding full length bovine CD40 (boCD40) was similarly generated. Recombinant swCD40ED was expressed as a FLAG-tagged protein by transfecting HEK-293 Free-Style cells (Invitrogen) and affinity purified using anti-FLAG M2-agarose affinity chromatography (Sigma) as previously described [30, 31].

### Monoclonal antibody production

Monoclonal antibodies (mAbs) against swCD40ED were produced as previously described [32]. Briefly, three female BALB/c mice were inoculated subcutaneously three times every 2 weeks with 50μg of recombinant swCD40ED in RIBI adjuvant (Sigma-Aldrich). The mice were housed under temperature controlled and lighting controlled conditions in specific pathogen-free (SPF) facility at Texas A&M University. Seroconversion was monitored on a weekly basis by ELISA using plates coated with the recombinant swCD40ED (100ng/well). The mouse with the best anti-swCD40ED antibody response was sedated by isoflurane and then stimulated by retroorbital injection of 50μg of the recombinant swCD40ED without adjuvant. Three to Five days after retro orbital injection, the mouse was euthanized by CO_2_ and the spleen was harvested. On the day of fusion, the spleen was harvested for preparing single cell suspension for electrofusion with Sp2/0 myeloma cells (ATCC, Manassas, VA). Hybridomas were plated in 96well cell culture plates (Nunc) and grown in hypoxanthine-aminopterin-thymidine (HAT) medium. Primary screening was performed by ELISA on day 14 post-fusion using ELISA plates coated with recombinant swCD40ED as above. Proliferation assay was used to test ELISA positive hybridomas for agonistic effect on swine PBMCs. Positive hybridoma clones identified by proliferation assay were subcloned by limiting dilution and retested by ELISA and proliferation assays. The leading candidate, clone 2E4E4, was isotyped using the Mouse Immunoglobulin Isotyping ELISA Kit (BD Pharmingen) following the manufacturer’s protocol and was selected for further analysis.

### Immunocytometric analysis

HEK-293A cell monolayers were transfected with constructs encoding either swCD40 (pcDNAswCD40) or boCD40 (pcDNAboCD40) using Polyethylenimine (Polyscience) as previously described [33]. Following 48hr. incubation, the monolayers were fixed with cold methanol, rinsed with PBS, blocked with 10% FBS/PBS solution, and incubated for 1 hr. at room temperature with 5μg/mL of the mAb 2E4E4 or 5μg/mL of an IgG1 isotype control (Biolegend). The cell monolayers were washed 3X with blocking buffer and then incubated for 1 hr. with Alkaline Phosphatase AffiniPure F(ab’)_2_ fragment donkey anti-mouse IgG (H+L) (Jackson ImmunoResearch Laboratories, INC). Following washes as above, Fast Red TR–Naphthol AS-MX substrate (Sigma, F4523) was used to detect alkaline phosphatase activity. Photos were captured using Spot RT3 camera on Olympus IX70 microscope.

### Flow cytometry

#### Transfection of HEK-293A cells

The pcDNAswCD40 and pcDNAboCD40 constructs were used to transfect HEK-293A cell monolayers using Polyethylenimine (Polyscience) as previously described [33]. Following 48 hr. incubation, one million transfected cells were added to each well of a 96 well V-bottom plate and stained with Zombie Red^TM^ Fixable Viability Kit (Biolegend) following the manufacture's protocol. The pcDNAswCD40 and pcDNAboCD40 transfected HEK-293A cells were incubated with 5μg/mL of the mAb 2E4E4 or 5μg/mL of IgG1 isotype control for 30 min. and washed 3X with blocking buffer (cDMEM with 0.05% sodium azide/20% bovine Serum). The cells were incubated for 30 min. with AffiniPure F(ab’)_2_ Fragment Donkey Anti-Mouse IgG (H+L) conjugated with FITC (Jackson ImmunoResearch Laboratories, INC.), washed 3X with block buffer, and stored in FACS fixer (12.5% formaldehyde/PBS). Data was collected using BD FACScalibur™ (Becton Dickinson) and data analysis was done using FlowJo 10 software (FlowJo).

#### LPS stimulated swine and bovine PBMCs

A minimum of 30mL of blood was collected from pigs, cows, goats, and sheep. The blood was then processed to isolate PBMCs by Histopaque® (Sigma-Aldrich) density gradient centrifugation following the manufacturer’s protocol. Swine and bovine PBMCs were added to a 12-well plate (4 million PBMCs per well) and incubated for 24 hr. in 1mL of complete RPMI alone or in cRPMI containing LPS (10μg/mL). Half a million swine or bovine PBMCs from either treatment was added to each well of a 96 well V-bottom plate (Axygen), stained with Zombie red^TM^ viability kit (Biolegend) following manufacturer’s protocol, and blocked using either swine blocking buffer (cRPMI with 0.05% sodium azide/20% swine serum) or bovine blocking buffer (cRPMI with 0.05% sodium azide/20% bovine serum). The swine and bovine PBMCs were incubated for 30 min. on ice with either 5μg/mL of the mAb 2E4E4 conjugated to FITC or 5μg/mL of IgG1 isotype control (Biolegend) conjugated to FITC. The antibodies were conjugated to FITC using FluoroTag^™^ FITC Conjugation Kit (Sigma-Aldrich) following manufacturer’s protocol. After incubation with the mAbs, the cells were washed 3X with blocking buffer, and fixed using FACS Fixer. Flow cytometry data was collected and analyzed as above.

#### MHC class II expression

Swine PBMCs were seeded in a 12-well plate at 4 million PBMCS per well in 1mL of cRPMI media alone, media containing LPS (10μg/mL), or media containing graded amounts of the mAb 2E4E4 (0.5, 1.0, 2.5, 5.0μg/mL). After a 24-hr. incubation, half a million PBMCs were added to each well of a 96 well V-bottom plate (Axygen), blocked (20% swine serum/FACS media) for 30 min. on ice, and then incubated for 30 min. with 5μg/mL of mouse anti-swine MHCII-FITC clone MSA3 (Monoclonal Antibody Center Washington State University). The PBMCs were washed 3X with blocking buffer, and then fixed and stored in FACS fixer. Flow cytometry data was collected and analyzed as above.

### Immunohistochemistry

Swine, bovine, caprine, ovine, feline, erinaceine, and cavy spleen tissue (donated by Texas A&M Veterinary Medical Diagnostic Laboratory) were used to prepare histology slides as previously described [34]. A Intellipath (Biocare Medical) automatted immunohistochemistry slide staining system was used to stain the slides by following the protocol described below. The slides were incubated for 20 min. with Peroxidazed 1 (Biocare Medical), washed with TBS 1X for 15 secs, and incubated with Background Sniper (Biocare Medical) for 20 min. After washing as described above, the slides were incubated with 5μg/mL of the mAb 2E4E4 or 5μg/mL of IgG1 isotype control. Following 1 hr. incubation, the slides were washed 1X with TBS and incubated for 1 hr. with Immpress^TM^ Goat Anti-Mouse IgG Serum-HRP (Vector) secondary antibody. After washing the slides as described above, Horseradish peroxidase activity was tested using Nova Red (Vector Labs) and then counter-stained with crystal violet.

Photos were captured using Spot RT3 camera on Olympus IX70 microscope.

### Proliferation assay

Swine (Yorkshire), bovine (Holstein and Black Angus), caprine (Boer Spanish cross), and ovine (Scottish Blackface) PBMCs (250,000 cells/well) were cultured in triplicate wells of 96 well round bottom plates for 24 hr. in a total volume of 100μL of cRPMI containing graded amounts of either mAb 2E4E4) or IgG1 isotype control (0.5, 1.0, 2.5, 5.0, or 10 μg/mL), PMA (1μg/mL)/Ionomycin (0.5μg/mL), or media alone. The cells were labeled for 12 hr. with 0.3 μCi of ^3^H-thymidine and incorporation of the isotope (in counts per minute) by the cells was determined using Microbeta Counter (PerkinElmer). Stimulation index (SI) was calculated from the CPM data for both 2E4E4 and IgG1 isotype control by dividing the treatment (2E4E4 or IgG1 isotype control) counts by the media control counts.

### Nitric oxide assay

The level of Nitrite (NO_2_^-^) released by activated bovine monocyte-derived macrophages was measured by Griess assay as previously described [35]. Briefly, the macrophages (200,000 cells/well) were added in triplicate wells of a 96 well flat bottom plate containing graded amounts of either mAb 2E4E4 or IgG1 isotype control (0.5, 1.0, 2.5, 5.0, 10μg/mL), LPS (10μg/mL), or media alone. Following a 24-hr. incubation, macrophage supernatants were tested for Nitrite concentration using Nitric Oxide Assay kit (ThermoFisher) following manufacturer’s protocol. Nitrite released was presented as μM NO_2_^-^.

### Intracellular cytokine staining

For intracellular cell cytokine staining, swine PBMCs (4 million cells) were added to each well of a 12 well plate containing graded amounts of the mAb 2E4E4 (1.0, 2.5μg/mL), LPS (1μg/mL) or media alone. The cells were incubated for either 12 or 24hr., and 12hr before cells were harvested, Brefeldin A was added to each well. The PBMCs were plated in 96 well v-bottom plate (5 x 10^5^ cells/well), incubated for 15 min. in PERM/WASH™, blocked (20% porcine serum in 1X PERM/WASH™ buffer), and further incubated with 5μg/mL of mouse anti-swine TNF-α clone 103314 (R&D Systems) conjugated with FITC, mouse anti-swine IL-1α clone 85733.11 (R&D Systems) conjugated with FITC, mouse anti-swine IL-6 clone 77830 (R&D Systems) conjugated with FITC, or mouse anti-swine IL-8 Clone 105115 (R&D Systems) conjugated with FITC for 1hr. After 3 washes with blocking buffer, the cells were fixed and stored in FACS fixer. Flow data was collected, and analyzed as above.

### Statistics

All analyses were performed using GraphPad 6.05 software. Data from the Nitric oxide assay was analyzed by two-way ANOVA with Sidak's multiple comparisons test comparing similar concentrations of 2E4E4 to IgG1 isotype control. The proliferation assay data was analyzed by two-way ANOVA with post hoc Tukey’s multiple comparisons tests comparing similar concentrations of 2E4E4 to IgG1 isotype control. Intracellular cytokine data was also analyzed by two-way ANOVA with Dunnett’s test multiple comparisons by comparing the media control to the treatment from the same time point. MHC-II flow cytometry data was tested for correlation between PBMCs incubated with mAb 2E4E4 and by the MHC-II positive percentage by performing a one-tailed Spearman correlation test. A value of p≤0.05 was considered0020statistically significant.

### Ethics statement

The study was conducted in accordance with the Public Health Service Policy on Humane Care and Use of Laboratory Animals as specified in the Health Research and Extension Act of 1985 (Public Law 99–158) or in accordance with the U.S Department of Agriculture policies as required by the Animal Welfare Act of 1966 (7.USC.2131 *et seq*) as amended in 1970, 1976, and 1985. The research protocol: AUP 2013–0092 was reviewed and approved by the Texas A&M University Institutional Animal Care and Use Committee to ensure compliance with PHS standards. All animal care facilities are inspected twice per year.

The facilities and procedures for maintenance and care of animals are accredited by the American Association for Accreditation of Laboratory Animal Care. Efforts were made to minimize suffering, and at the completion of the study, the mice were euthanized by CO_2_ narcosis followed by cervical dislocation. This method is approved by the Panel on Euthanasia of the American Veterinary Medical Association. Porcine, bovine, ovine, caprine, canine, feline, cavy, and erinaceine tissues and cells were acquired from the Texas A&M University tissue share program. Porcine, bovine, ovine, and caprine tissues and cells were acquired from the Texas A&M University tissue share program.

## Results and Discussion

### The mAb 2E4E4 recognized cell-surface expressed CD40

**Fig 1:**
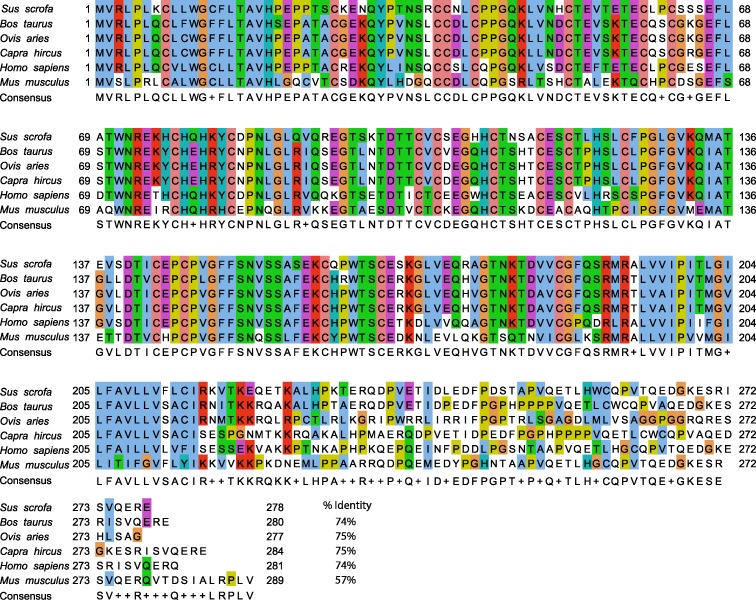
Bovine, ovine, and caprine CD40 protein sequences have high homology to swine CD40 protein sequence. Alignment of swine, bovine, caprine, and ovine CD40 amino acid sequences. The signal sequence is shown where the consensus sequence is highlighted in green (amino acid 1–19), whereas the consensus sequence of the transmembrane domain is highlighted in red (amino acid 192–215). The percentage identity of the extracellular domains of bovine, ovine, and caprine CD40 protein sequences to that of swine is 74%, 75%, and 75%, respectively.

**Fig 2:**
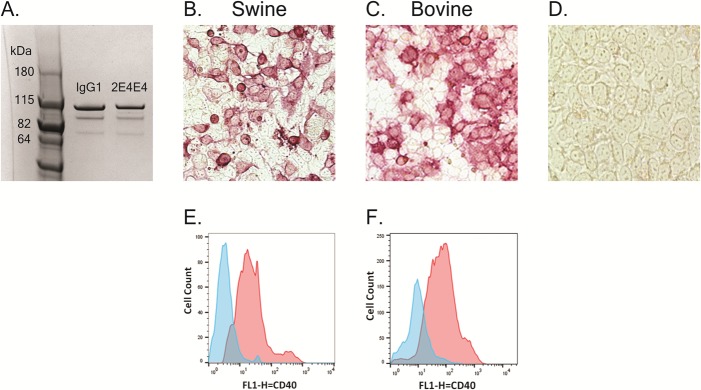
Reactivity of the mAb 2E4E4 against HEK-293A cells expressing swine or bovine CD40. Evaluation of the mAb 2E4E4 specificity against swine and bovine CD40 was performed by immunocytometric analysis: A. Analysis of mAb 2E4E4 and a defined IgG1 control mAb by PAGE; B. HEK-293A cells transfected with a construct expressing full length swine CD40 and probed with 2E4E4; C. HEK-293A cells transfected with a construct expressing full length bovine CD40 and probed with the mAb 2E4E4; and D. Sham treated HEK-293A cells probed with the mAb 2E4E4. Flow cytometric analysis performed on: E. HEK-293A cells transfected with a construct encoding full length swine CD40; or F. full length bovine CD40 probed with either the mAb 2E4E4 (Red) or IgG1 isotype control (Blue).

A mouse anti-swine CD40 mAb, designated 2E4E4, was generated by immunizing mice using recombinant extracellular domain of swine CD40 (Fig 1). The isotype of mAb 2E4E4 was determined to be IgG1_k_. Immunocytometric analysis of HEK-293A cells transfected with a construct expressing full length swine CD40 confirmed that the mAb 2E4E4 recognized CD40, whereas sham treated cells were negative (Fig 2B and 2D). This outcome was also confirmed by performing flow cytometry on similarly transfected cells. The mAb 2E4E4, but not IgG1 isotype control, strongly recognized surface-expressed swine CD40 (Fig 2E). Since swine and bovine CD40 protein sequences are highly conserved (Fig 1), we predicted that mAb 2E4E4 could also bind to bovine CD40. Indeed, immunocytometric and flow cytometric analysis of HEK-293A cells transfected with a construct expressing full length bovine CD40 yielded similar results (Fig 2C and 2F). These outcomes showed that the mAb 2E4E4 could bind to both swine and bovine CD40, and hence all further experiments were designed to evaluate the interaction of the mAb 2E4E4 with bovine CD40 as well.

### The mAb 2E4E4 recognized native swine and bovine CD40

**Fig 3:**
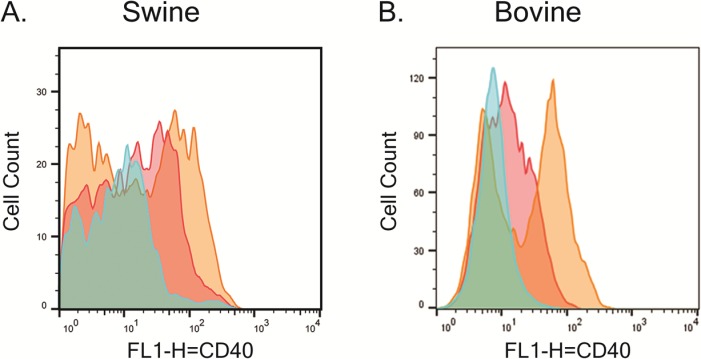
The mAb 2E4E4 recognized CD40 on stimulated swine and bovine PBMCs. Flow cytometry performed on A) swine and B) bovine PBMCs stimulated (Gold) or not stimulated (Red) with LPS and probed with the mAb 2E4E4. IgG1 isotype control (Blue) was also used to probe LPS-stimulated swine and bovine PBMCs.

**Fig 4:**
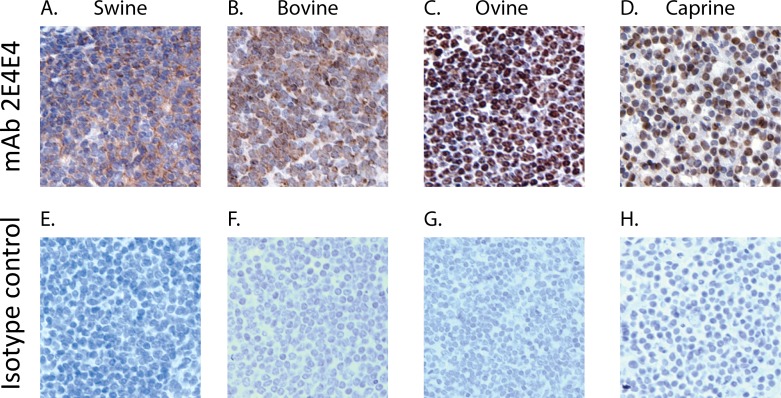
Validation of the specificity of the mAb 2E4E4 against CD40 expressed in swine, bovine, ovine, and caprine spleen. Immunohistochemistry performed on: A) swine; B) bovine; C) ovine; and D) caprine spleen tissues probed with the mAb 2E4E4. Background reactivity was tested by probing E) swine, F) bovine, G) ovine, and H) caprine spleen tissues with an IgG1 isotype control mAb.

Flow cytometric analysis showed that the mAb 2E4E4 bound to the CD40 expressed on LPS stimulated swine and bovine PBMCs. The mAb 2E4E4 showed fluorescence on stimulated PBMCs as compared to non-stimulated controls. A specific signal was also detected on nonstimulated PBMCs probed with the mAb 2E4E4, but not on cells probed with an IgG1 isotype control (Fig 3). These results showed that the mAb 2E4E4 bound to the CD40 expressed on the cell surface of stimulated PBMCs. These outcomes are consistent with previous findings which showed that PBMCs upregulate CD40 on their cell surface when stimulated with LPS [36]. In addition, immunohistochemistry (IHC) showed that mAb 2E4E4, but not an IgG1 isotype control, reacted strongly to swine and bovine spleen tissues (Fig 4A, 4B, 4E and 4F). Taken together, the flow cytometry and IHC data showed that the mAb 2E4E4 recognized CD40 expressed on swine and bovine cells. In addition, IHC data also showed cross-reactivity of the mAb 2E4E4 to caprine and ovine CD40 (Fig 4C and 4D). Surprisingly, staining was also observed on spleen tissues from feline, cavy, and erinaceine and lymph node tissue from canine (S2 Fig). These results surprised us as cavy and erinaceine protein sequences had less than 65% identity to swine CD40, while the other species had CD40 protein sequences around 70% or more identity. CP 870,893 is an agonistic anti-human CD40 mAb that is currently being used in clinical trials. Interestingly, CP 870,893 did not show any agonistic affect against mice, rat, rabbit, or dog cells, while mAb 2E4E4 was able to bind to canine cells [37]. This data shows the unique species range mAb 2E4E4 can recognize. Although mAb 2E4E4 was shown to have cross reactivity against multiple species, it did not react with human macrophage cell lines but studies with primary human cells are yet to be done. Given this outcome, it is likely that mAb 2E4E4 recognizes a CD40 determinant that is distinct from the one recognized by anti-human CD40 mAbs. This outcome is supported by the observation that no anti-human CD40 mAbs cross-reacts with swine CD40.

### Agonistic effect of mAb 2E4E4

**Fig 5:**
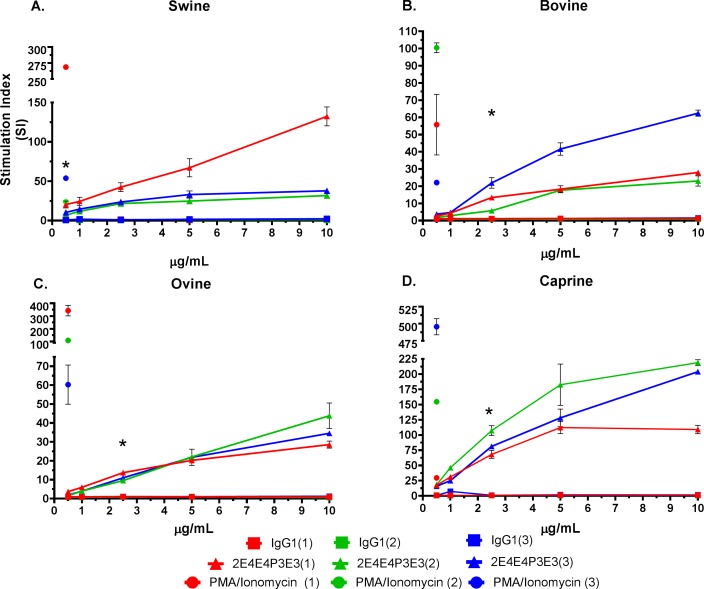
Stimulation of swine and bovine PBMC proliferation by the mAb 2E4E4. Agonistic activity of the mAb 2E4E4 on swine, bovine, ovine, and caprine PBMCs was evaluated by ^3^H-Thymidine incorporation. Panels: A) swine, B) bovine, C) ovine, and D) caprine PBMCs responses after incubation with 2E4E4 (representative data is shown for 3 animals from each species) or an IgG1 isotype control. Each point represents the mean SI from triplicate wells ± SD; *Significant (p<0.001) mAb 2E4E4-induced proliferation of PBMCs from all the three 3 animals compared to IgG1 isotype control treatment.

**Fig 6:**
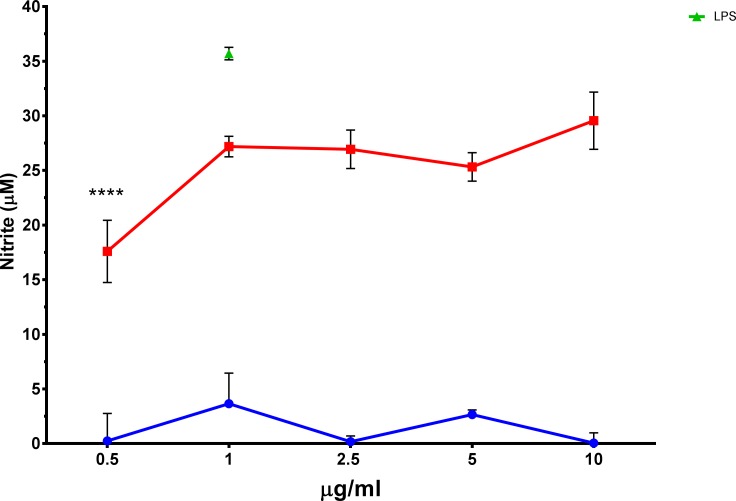
Stimulation of Nitric oxide production by mAb 2E4E4. Agonistic effect of 2E4E4 was verified by nitric oxide assay using bovine monocyte-derived macrophages incubated with graded amount of the mAb 2E4E4 (Black) or IgG1 isotype control mAb (Gray). Each column represents the μM of NO_2_^-^ mean of triplicate wells stimulated with the mAb 2E4E4 at each concentrations ± SD; n = 3; * p<0.0001.

The mAb 2E4E4 showed significant agonistic effects on swine, bovine, caprine, and ovine PBMCs. The mAb 2E4E4 stimulated significant (p<0.001) proliferation of swine and bovine PBMCs compared to the IgG1 isotype control (Fig 5A and 5B). Interestingly, the mAb 2E4E4 also had significant stimulatory (p<0.001) effect on ovine and caprine PBMCs in a dose dependent manner (Fig 5C and 5D). The stimulatory effect is consistent with previous findings that showed that an agonistic anti-human CD40 mAb named CP 870,893 activated proliferation of human lymph node cells. Further evidence of the agonistic effect of mAb 2E4E4 was shown by demonstrating that the mAb, but not an IgG1 isotype control, stimulated significant (p<0.0001) NO release by bovine macrophages (Fig 6). We also tested NO production by swine macrophages, but the outcome was negative. Previous studies showed that swine macrophages stimulated with agonists, such as LPS, do not transcribe nitric oxide synthase mRNA and thus do not synthesize nitric oxide, and this outcome is consistent with our observation [38–40].

**Fig 7:**
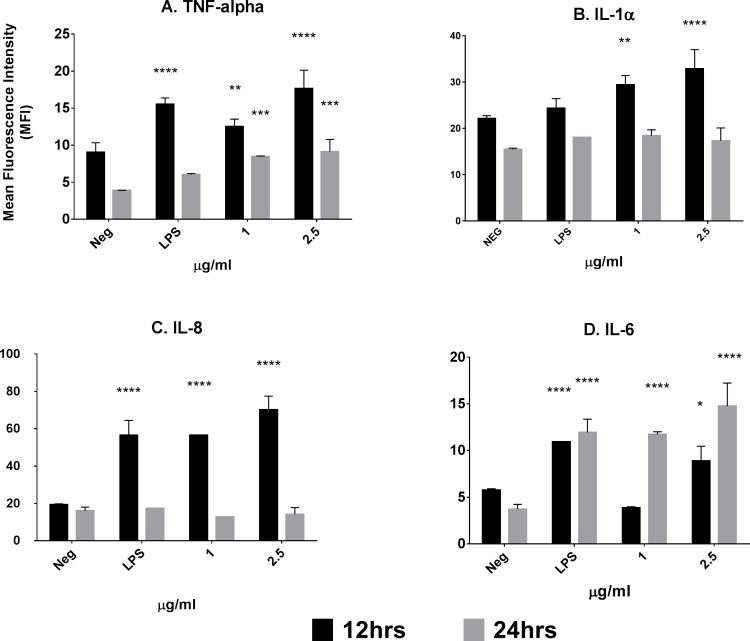
Upregulation of pro-inflammatory cytokine response by mAb 2E4E4. Intracellular cytokine staining was used to evaluate the ability of mAb 2E4E4 to stimulate upregulation of pro-inflammatory cytokines. Swine PBMCs were incubated with the mAb 2E4E4, LPS, or media alone, harvested at 12 hr. (Gray) and 24 hr. (Black), and then probed with mAbs against A) IL-1α; B) TNF-α; C) IL-6; or D) IL-8. Each column represents the mean florescent intensity of two wells ± SD. *p<0.05.

Flow cytometry confirmed that the mAb 2E4E4 upregulated MHC-II expression on swine PBMCs. A significant increase (p<0.01) in MHC-II expression was observed when swine PBMCs were stimulated with the mAb 2E4E4 and the response was dose dependent (Table 1). The MHC-II upregulation is expected to result in enhanced antigen presentation by APCs as has previously been shown [41, 42]. The agonistic effect of mAb 2E4E4 was further investigated by intracellular cytokine staining. Significant (p<0.01) upregulation of TNF-α, IL-1α, and IL-8 was observed at 12hr. post-stimulation (Fig 7A–7C). No significant expression of IL-1 α or IL-8 was detected after incubating the PBMCs with mAb 2E4E4 for 24 hr. Significant (p<0.001) expression of TNF-α was observed at 24 hr. but at a lower level of expression than seen at 12hr. Unlike TNF-α, IL-1α, and IL-8, significant (p<0.001) IL-6 expression was observed for 1μg/mL of mAb 2E4E4 after 24hr. post-stimulation (Fig 6D). PBMCs incubated with 2.5 μg/mL of mAb 2E4E4, however, had significantly (p<0.05) upregulated IL-6 at 12hr. and continued to upregulate IL-6 at 24 hr. This delay in expression of IL-6 is consistent with previous findings [43, 44]. Upregulation of MHC-II and pro-inflammatory cytokines have previously been shown to be important signals for stimulation of an adaptive immune response against a vaccine. These studies have reported that MHC-II signaling is required for CD4^+^ T-cell and APCs activation, while pro-inflammatory cytokines such as IL-6 are important for activating the immune system and modulating TH1/TH2 responses [45–47]. Our results showed that the mAb 2E4E4 can stimulate upregulation of MHC-II and release of pro-inflammatory cytokines, thus suggesting that the mAb 2E4E4 has several activities that would be beneficial as a vaccine adjuvant.

**Table 1:** MHC-II upregulation by swine PBMCs stimulated with the mAb 2E4E4.

Sample	MHCII % positive cells
**LPS**	19.0
**0 μg/mL (2E4E4)**	8.77
**0.5 μg/mL (2E4E4)**	11.9
**1 μg/mL (2E4E4)**	14.9
**2.5 μg/mL (2E4E4)**	16.6
**5 μg/mL (2E4E4)**	22.2

Swine PBMCs were incubated with the mAb 2E4E4 or LPS for 24hr. and MHC-II upregulation was determined by flow cytometric analysis. Data is presented as absolute percentage of MHCII positive cells compared to IgG1 isotype control.

In summary, an anti-swine CD40 mAb, designated 2E4E4, was generated, characterized and demonstrated to be cross-reactive to bovine, ovine and caprine CD40. The mAb 2E4E4 was shown to specifically bind to CD40 on swine and other species including bovine, caprine, and ovine. In addition to the mAb 2E4E4 having agonistic effects on swine cells, it also showed broad agonistic effects against bovine, caprine, and ovine cells. These outcomes suggest that mAb 2E4E4 has a potential for development of a broad immune modulator for use in livestock. Agonistic anti-CD40 mAb is a more attractive stimulant for adjuvant development compared to other CD40 agonist such as recombinant CD40L or C_3_-sysmmetric complex since, a previous study that utilized these agonists reported that agonistic anti-mouse CD40 mAb (3/23) had a 2fold increase in B-cell proliferation compared to CD40L. While C_3_-sysmmetric complex showed no agonistic activity when added to mouse PBMCs, it had synergistic effect when mixed with agonistic anti-CD40 mAb [48]. Immune modulators, such as CP-870,893 (an anti-human CD40 mAb), recently been shown to be useful in treating a broad range of cancers and function as a strong adjuvant component [49, 50]. Patients who have received agonistic anti-CD40 have shown an increase in memory CD8^+^ CTLs, CD27^+^ memory B-cells, and CD27^+^/CD86^+^ memory Bcells [51, 52]. An adjuvant that increases memory B-cells and CD8 memory T-cells would potentiate vaccines to confer better immune protection against broad range of diseases. Unlike most of the immune modulators that are currently in use, a broad spectrum immune modulator, such as mAb 2E4E4, that can be used in a variety of animals will be a valuable resource in the development of new treatments and vaccines for livestock diseases [37].

## Supporting Information

S1 Fig Feline, canine, erinaceine, and cavy CD40 protein sequences have homology to swine CD40 protein sequence. Alignment of feline, canine, erinaceine, and cavyCD40 amino acid sequences. The signal sequence is shown where the consensus sequence is highlighted in green (amino acid 1–19), whereas the consensus sequence of the transmembrane domain is highlighted in red (amino acid 192–215). The percentage identity of the extracellular domains of feline, canine, erinaceine, and cavy CD40 protein sequences to that of swine is 75%, 70%, 64% and 65%, respectively.
(TIF)

S2 Fig Validation of the specificity of the mAb 2E4E4 against CD40 expressed in swine, feline, canine, erinaceine, and cavy. Immunohistochemistry performed on: A. feline C. cavy D. erinaceine spleen and B. canine Lymph node tissues probed with the mAb 2E4E4. Background reactivity was tested by probing E. feline, G. cavy, and H. erinaceine spleen and F. canine lymph node tissues with an IgG1 isotype control mAb.
(TIF)
