# Supplementary figures and images for: Characterization of a Broadly Reactive Anti-CD40 Agonistic Monoclonal Antibody for Potential Use as an Adjuvant
Source: PLoS One. 2017 Jan 20;12(1):e0170504. doi: 10.1371/journal.pone.0170504 (PMC5249191; doi:10.1371/journal.pone.0170504)

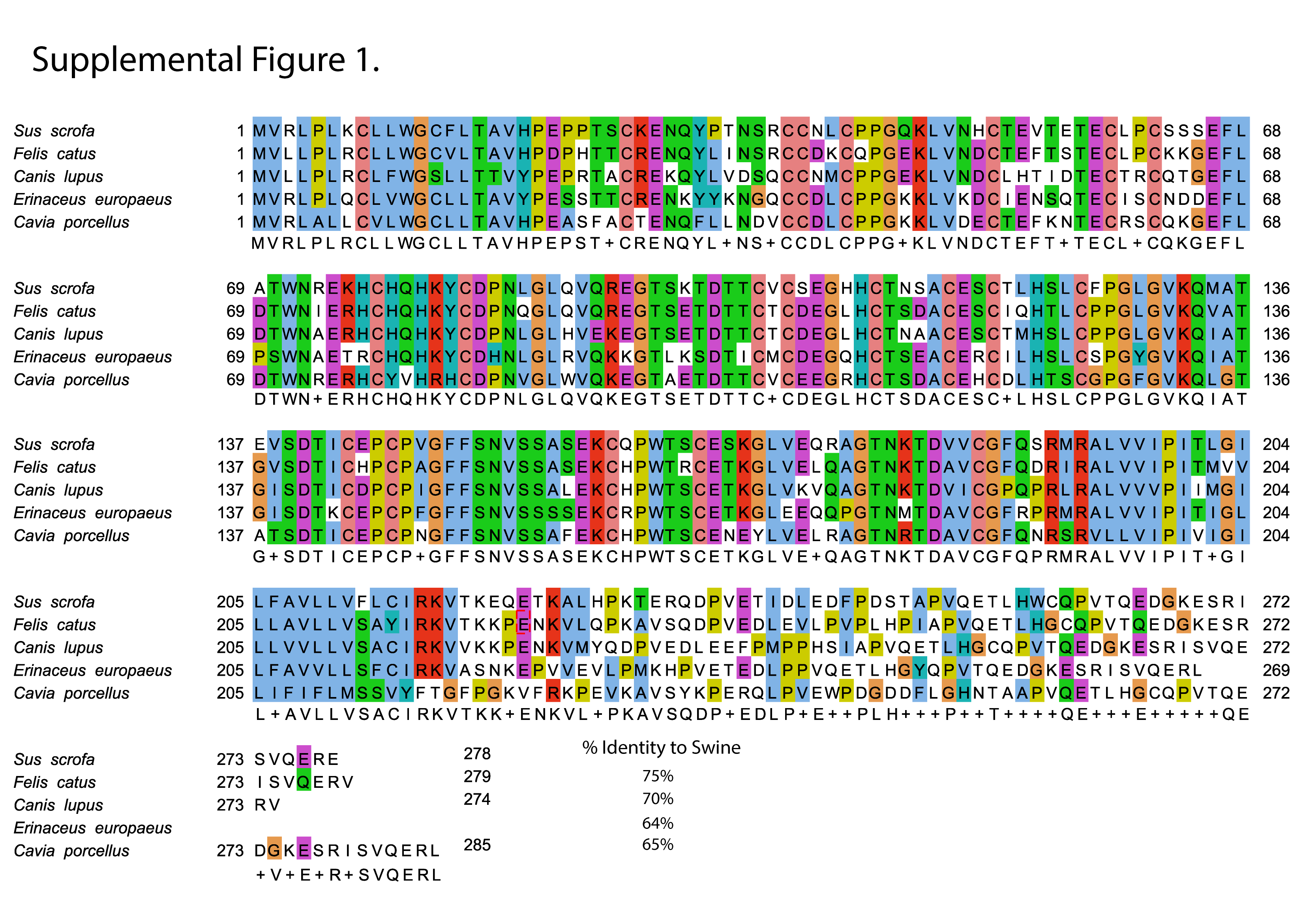

Supplement: S1 Fig — Alignment of feline, canine, erinaceine, and cavyCD40 amino acid sequences. The signal sequence is shown where the consensus sequence is highlighted in green (amino acid 1–19), whereas the consensus sequence of the transmembrane domain is highlighted in red (amino acid 192–215). The percentage identity of the extracellular domains of feline, canine, erinaceine, and cavy CD40 protein sequences to that of swine is 75%, 70%, 64% and 65%, respectively. (TIF) [file pone.0170504.s001.tif]

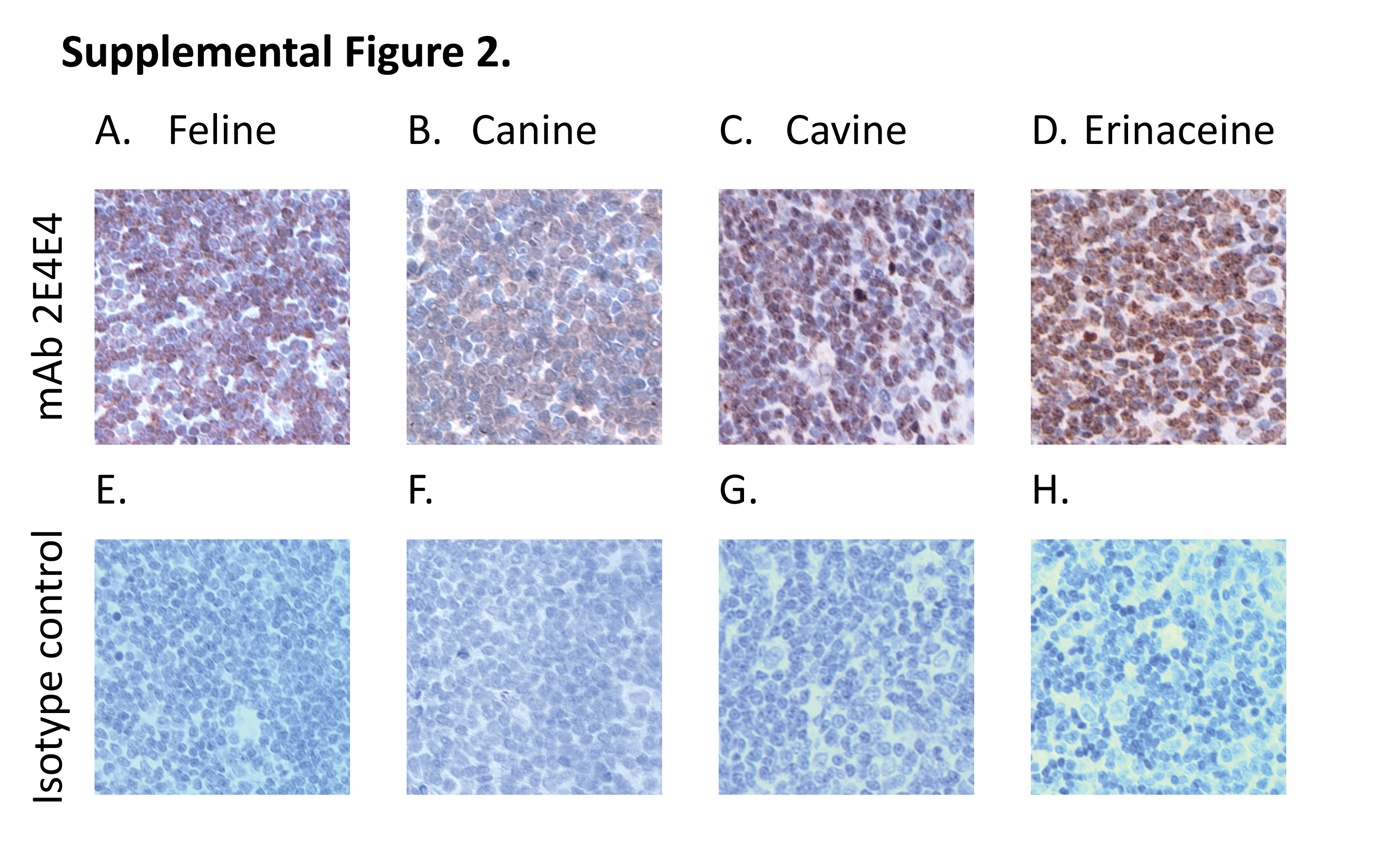

Supplement: S2 Fig — Immunohistochemistry performed on: A. feline C. cavy D. erinaceine spleen and B. canine Lymph node tissues probed with the mAb 2E4E4. Background reactivity was tested by probing E. feline, G. cavy, and H. erinaceine spleen and F. canine lymph node tissues with an IgG1 isotype control mAb. (TIF) [file pone.0170504.s002.tif]
